# Immunocap ISAC as an important diagnostic tool in rhino-sinusitis

**DOI:** 10.1186/2045-7022-3-S2-P32

**Published:** 2013-07-16

**Authors:** Michael Rudenko, Suranjith Seneviratne, Robert Boyle, Katya Burova, William Egner, Amina Bhayat-Cammak, Neal Bradshaw

**Affiliations:** 1 London Allergy and Immunology Centre, Outpatient Clinic, London, UK; 2 Protein Reference Unit Sheffield Teaching Hospitals, Immunology, London, UK; 3 Protein Reference Unit Sheffield Teaching Hospitals, Immunology, Sheffield, UK; 4 Thermo Fisher Scientific, ImmunoDiagnostics, London, UK

## Background

Recent developments in immunology have started to influence clinical allergy by introducing a promising approach of molecular allergy (allergology) - ImmunoCap ISAC.

## Aim

We present a unique case of severe allergic rhino-sinusitis in a 39 y.o. female patient that was diagnosed using ISAC.

## Method

ISAC is designed to detect specific IgE antibodies to a large number of allergenic epitopes **–** components of known allergens from a single blood test.

## Results

The patient (smoker) complained on a variety of symptoms that were bothering her for more than 3 years: headaches, significant fatigue, nasal blockage, breathlessness on exercise and occasionally at rest. There were no ocular symptoms. She had several courses of antibiotics during this period with slight improvement. Careful history revealed progressive deterioration in her condition; there was no difference in her symptom pattern through the season although she noted that she had more sinus pains in winter. There was a dog, but no dampness in the house. There was no personal or family history of atopy. Anterior rhinoscopy had appearance of acute inflammation with no structural abnormalities. Spirometry showed FEV1 95%, FVC 92% and Peak flow 85% predicted. Skin prick test with valid controls, was negative to a standard panel of aeroallergens including dog. Immunocap ISAC test was offered to broaden the spectrum of allergens tested. This showed - mono-sensitisation to Dog rCan f 5 Arginine esterase 1,5 ISU and negative to all other components.

## Conclusion

The spectrum of usually tested dog allergens appears incomplete: Two lipocalins, Can f 1 and Can f 2, and serum albumin, Can f 3, have been characterised in detail but do not fully account for the IgE antibody-binding activity in all dog-allergic patients. Allergen activity has previously been detected in dog urine Can f 5, believed to be produced only in male dogs. ISAC was very beneficial in this case and opened a new insight on the diagnostic process.

